# Reliability of the Athlete Food Choice Questionnaire in Diverse Settings

**DOI:** 10.3390/ijerph19169981

**Published:** 2022-08-12

**Authors:** Rachael L. Thurecht, Fiona E. Pelly, Sarah Burkhart

**Affiliations:** School of Health and Behavioural Sciences, University of the Sunshine Coast, Maroochydore, QLD 4558, Australia

**Keywords:** determinant, food preferences, athletic performance, sports, exercise, nutrition support

## Abstract

Understanding the factors that influence an athletes’ food choice is important to supporting optimal dietary intake. The Athlete Food Choice Questionnaire (AFCQ) is a new validated tool for assisting practitioners and researchers to understand athlete eating behaviours. However, the AFCQ previously has only been applied at international competition events. This observational study explored the online application of the AFCQ outside of the competition environment with detailed examination of factor reliability. The AFCQ factors include ‘nutritional attributes of the food’, ‘emotional influences’, ‘food and health awareness’, ‘influence of others’, ‘usual eating practices’, ‘weight control’, ‘food values and beliefs’, ‘sensory appeal’, and ‘performance’. A total of 131 athletes, representing 19 countries and 36 sports, participated using an online questionnaire. Reliability via Cronbach’s alpha (α) and item correlation scores were compared to those from previous competition events. Acceptable reliability was attained for seven of the nine factors (α ≥ 0.7, range 0.70–0.92). ‘Food values and beliefs’ and ‘usual eating practices’ (α = 0.60, 0.64) were tolerable and consistent against previous major competition samples, indicating that the setting is unlikely responsible for lower reliability scores. Three factors (‘emotional influence’; ‘nutritional attributes of the food’; ‘influence of others’) differed in reliability between the online sample compared to one or both major competition samples. The ‘religious food beliefs’ item within the ‘food values and beliefs’ factor may warrant removal due to recurrent low internal consistency. This study confirms the AFCQ’s reliability regardless of competition phase and supports use of the AFCQ for understanding the eating behaviours of athletes.

## 1. Introduction 

An athlete’s dietary intake is influenced by a multitude of factors that are potential enablers or barriers to appropriate food selection [1,2,3,4]. An optimal dietary intake is important for health and performance; thus, an understanding of the key factors impacting athletes’ food choices is necessary for providing effective nutrition support. A recent scoping review highlighted many gaps in the understanding of the determinants of athletes’ food choices [5]. The new Athlete Food Choice Questionnaire (AFCQ) was developed to examine the influence that a variety of factors have on athletes’ food choices with applicability both during training and competition [6,7].

The AFCQ captures the frequency of influence that 32 items across 9 factors have on an athlete’s food choices (be that a single meal or an individual food or beverage). Development and validation was via a two-step process of principal component analysis (PCA) and confirmatory factor analysis (CFA) from diverse samples of athletes competing at the 2017 Universiade, Taiwan [6], and 2018 Commonwealth Games, Australia [7]. Despite differences in the demographic characteristics and sport representation of the two samples, construct validity was established through duplicate methods of both discriminant and convergent validity, resulting in nine and six factors, respectively, meeting all acceptable thresholds [7]. The final validated AFCQ factors are ‘nutritional attributes of the food’, ‘emotional influences’, ‘food and health awareness’, ‘influence of others’, ‘usual eating practices’, ‘weight control’, ‘food values and beliefs’, ‘sensory appeal’, and ‘performance’. Given that the sample of athletes were in varying competition stages (pre, between, and post their competition event/s), the stability of the factorial structure supports the applicability of the AFCQ for use with athletes across broader training and competition phases.

Reliability is important to establish for new survey tools to ensure the items within a factor are intercorrelated and measuring the same underlying construct [8]. Commonly, reliability of survey tools is measured via test–re-test to examine stability over time, Cronbach’s alpha score, and item intercorrelations to understand internal insistency [8]. The reliability of the AFCQ has been demonstrated to be acceptable for seven of the nine factors in a previous study [7]. The two factors ‘food values and beliefs’ and ‘usual eating practices’ were the least reliable in this study [7]. This may be attributable to inconsistent item responses according to athlete characteristics such as cultural background or nationality, gender, level of experience and the broader influences of their sport’s culture, and competition season [5]. In a descriptive analysis of the previous samples, ‘food values and beliefs’ was found to be rated more frequently as an influence by athletes from non-Western countries [9]. This suggests that further examination of the lower reliability factors against sample demographic characteristics is warranted.

Differences in reliability may also be attributed to the competition setting. During major competitions, athletes are often in a foreign country residing in an atypical food environment (e.g., athlete dining hall). An athlete village dining hall offers a wide range of convenient, free, prepared meals and foods that are easily accessible from a buffet service and are selected and eaten in the presence of peers, competitors, and coaches. Possible subconscious effects from the context of the setting environment have the potential to affect responses and therefore reliability for application in broader settings. As application of the AFCQ will more commonly occur outside of a major competition environment, it is valuable to re-examine internal consistency with athletes in standard phases of training and competition outside of a major event. Furthermore, the indicator items for the least reliable factors warrant investigation of internal consistency as well as examination of differences across demographic characteristics. The findings could assist those using, adapting, or interpreting the AFCQ for research and in practice.

The aim of this study was to (1) apply the AFCQ online to a diverse cohort of athletes outside of a major competition setting and (2) examine the reliability with comparison to previous samples of the nine AFCQ factors and internal consistency measures of indicator items within the ‘food values and beliefs’ and ‘usual eating practices’ factors. To aid in context of the reliability results and contribute to the literature on determinants of athlete food choices, a secondary study aim was to report descriptive outcomes the factors influencing athletes across demographic characteristics.

## 2. Materials and Methods

### 2.1. Measurement Instrument

This observational study applied the 32-item AFCQ plus 13 additional single items proposed by the authors [7] through an online questionnaire to domestic and international athletes. As the study aim was to compare reliability estimates, a diverse sample of athletes across sports and demographic characteristics were sought to reflect the diverse samples of the original validation studies [6,7].

The AFCQ question items were presented as neutral statements, and participants rated how frequently each item influenced their usual food choices (1 = never to 5 = always). Other factors that influence food choices were captured in an open-ended question. A summary of study variables and demographic characteristics collected is listed in Table 1. The additional food choice items on sodium content and fibre content excluded from the factor ‘nutritional attributes of the food’ during confirmatory factor analysis [7] were included in the present study due to their relevance on food choices for athletes in weight category [10] and endurance sports [11].

### 2.2. Data Collection

A convenience sample of athletes from a range of countries and sports was collected by dissemination of recruitment information in an e-newsletter for the international organisation for Professionals in Nutrition for Exercise and Sport (PINES). The study was also promoted through the authors’ professional networks by word of mouth and email. Snowballing recruitment and an incentive prize (two AUD 100 cash prizes drawn at random) were used to encourage participation. Participants who did not identify their role in sports as an athlete and those who were under 18 years old or reported their English capability at five or below were excluded from analysis. 

The questionnaire was administered electronically via Surveymonkey.com (February 2019–January 2021). All questionnaires were anonymous, and prize entry details were collected separately. A research information summary was provided on the first page of the online survey for potential participants to make an informed decision to participate. The research was conducted in accordance with the Declaration of Helsinki, with ethical approval granted by the Ethics Committee of the University of the Sunshine Coast (HREC no. 1/71/086).

The minimum sample size to detect Cronbach’s alpha at 0.70 is 21–24 for 5-item and 3-item factors, respectively, based on Bonett’s formula [12] and Bujang et al.’s summary tables [13]. A larger minimum sample size is needed when comparing Cronbach’s alpha reliability scores [13]. Assumptions used in the sample size calculation were 0.80 (CA_1_) Cronbach’s alpha as the average score from existing reliability data, a minimum of 3-items per factors for compared samples (k_1_ and k_2_), and a modest 80% power (β = 0.2). The minimum sample size of *n* = 126 is the mid-point between sample sizes calculated for estimated lower (0.65) and higher (0.90) score variations (CA_2_) (see supplementary for calculation).

### 2.3. Data Analysis

Composite scores (mean of item scores) for each factor are reported as median values (Mdn). The additional single items and specific indicator items were examined categorically after collapsing responses into three categories (never/rarely, sometimes, and often/always). Demographic characteristics were tested for association to the AFCQ factors via the Mann–Whitney U (U) and Kruskal–Wallis ANOVA (H) tests. Medians and non-parametric tests were utilized due to sample size and distributions on the limited response scale. Post hoc pairwise comparisons were conducted where significant results were found. Distributions between categorical variables examined by chi-square analysis and effect size identified using Cramér’s V (ϕc).

Sports were grouped into five physiology-based categories reported in previous literature [9,14,15]. The athletes’ countries of representation were grouped into seven regions based on geographic location and broad consideration of the region’s cultural style of eating. Consistent with similar studies [9,15,16], dichotomous categories for sport (individual and team) and country region (Western and non-Western) were used for statistical analysis. For the phase of competition question, ‘other’ responses pertaining to illness or injury were excluded, and the remaining responses were grouped into one of the three existing categories. Participants who indicated that their training was impacted by injury, illness, or recovery from surgery (*n* = 34) were excluded from tests when analysing the phase of competition variable to reduce potential confounding influence.

Reliability was assessed via Cronbach’s alpha and internal consistency scores (item-to-item and item-to-total correlations). Test–re-test analysis was not conducted, as the concept of factors influencing athlete food choices may not be stable over time due to variation of an athlete’s circumstances, such as where they are in their season or competitive cycle, how much training they are doing that day or week, if they are injured, etc., which may be difficult to account for and potentially introduce confounding bias. The thresholds for Cronbach’s alpha (α) were ≥0.6 minimally tolerable, ≥0.7 acceptable, and ≥0.8 good [8,17]. Results were compared to the reliability scores published within the AFCQ development [6] and validation studies [7]. Additional data from the development study was sourced from the authors to enable comparison of the item-to-total and item-to-item correlations (r) of the ‘food values and beliefs’ and ‘usual eating practices’ factors between samples. The web interface Cocron (version 1.0-1, accessed March 2021 from comparingcronbachalphas.org) was used for comparison of the Cronbach’s alpha coefficients, and pairwise comparisons were used to detect differences between samples [18]. Herein, the previous samples collected at the Universiade and Commonwealth Games are collectively referred to as the major competition samples.

The open-ended responses for other factors influencing participant’s food choices were examined by deductive content analysis utilizing a structured categorization matrix [19,20,21]. Responses were assigned descriptive codes, and responses that aligned with factors represented in the AFCQ’s 9 factors or the 13 additional items (simple factors) were excluded. The primary author conducted the content analysis, which was then reviewed by co-authors. 

Data analysis included use of Microsoft Excel (2013, Microsoft Corporation, Redmond, WA, USA) and Statistical Package for Social Sciences (SPSS) Statistical Software (version 26.0, IBM Corporation, Armonk, NY, USA). Missing data were addressed via listwise exclusion for statistical analyses. Significance threshold *p* < 0.05 and Bonferroni adjustment were applied to post hoc tests. The STROBE principals (Strengthening the Reporting of Observational studies in Epidemiology) [22] guided the preparation of this manuscript.

## 3. Results 

### 3.1. Descriptive Results 

A total of 343 responses were received; however, once ineligible responses were removed (*n* = 98 non-athletes, i.e., doctors, coaches; *n* = 89 incomplete responses; *n* = 19 under the age of 18 years; *n* = 4 duplicates; and *n* = 1 English capability not met), the final sample for analysis was 131 athletes. Participants represented 19 countries and 36 sports (Table 2). The mean age of participants was 27 ± 10.9 years (range 18–76 years, *n* = 128). The average score for participants’ confidence for reading and writing in English was M = 9.7 ±SD = 0.8 (range 6–10, *n* = 131). A total of 126 participants listed their language, with 83% (*n* = 105) reporting that they speak English at home.

There were significant differences between participant competition level and both region and prior nutrition education. A greater proportion of participants from Western countries had lower levels of competition history (57.7%, *n* = 56 national and state level) compared to participants from non-Western countries (17.6%, *n* = 6 national and state level; X^2^(2) = 17.9, *p* < 0.001). Athletes (*n* = 130) who had competed at higher levels of competition were more likely to have received most of their nutrition education from a nutritionist/dietitian or studied nutrition (47.6%, *n* = 39; X^2^(2) = 22.3, *p* < 0.001). Athletes (*n* = 130) with national or state level of competition history were more likely to receive the majority of their nutrition information from other sources (72.9%, *n* = 35; X^2^(2) = 22.3, *p* < 0.001). 

The factors with the highest median rating for food choice were ‘performance’, ‘food and health awareness’, and ‘sensory appeal’, while the least were the ‘influence of others’, ‘emotional influence’, and ‘food values and beliefs’. No significant differences were identified between factors according to participants’ phase of competition; however, multiple items differed according to participant characteristics for sex, sport, country region, and nutrition education (Table 3).

The ‘influence of others’ was rated higher as an influencing factor for athletes competing at the state, region, and national levels (Mdn = 3.00) compared to those who completed at other international competition levels (Mdn = 2.50, *p* = 0.029, H(2) = 6.73, *p* = 0.035, *n* = 130). Athletes that were trying to change their body weight or shape reported higher influence for the factors the ‘influence of others’ (Mdn = 3.00 vs. Mdn = 2.67, U = 2235.5, *p* = 0.017, *n* = 131) and ‘weight control’ (Mdn = 3.75 vs. Mdn = 3.13, U = 2479.0, *p* = 0.001, *n* = 131). 

‘Emotional influence’ differed according to the participant’s level of education (H(2) = 6.81, *p* = 0.033, *n* = 130). Athletes who had completed an undergraduate degree reported ‘emotional influence’ as a greater influence (Mdn = 3.00) than those who had completed senior school or lower levels of education (Mdn = 2.00, *p* = 0.029). ‘Emotional influence’ was higher rating for athletes who indicated that their training was impacted by injury, illness, or they were recovering from surgery (Mdn = 3.00 vs Mdn = 2.50, U = 2105.5, *p* = 0.016). 

**Figure 1:**
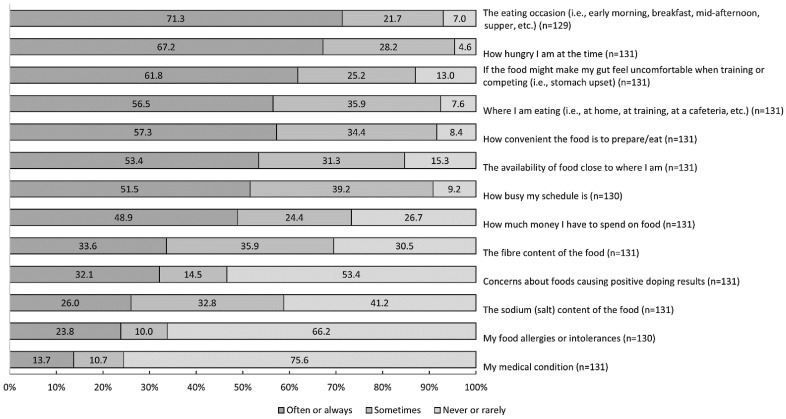
The proportion of 131 athletes rating how frequently (never/rarely, sometimes, and often/always) 13 individual items influence their food choices.

For the 13 additional items ‘eating occasion’ received the highest proportion (28.7%, *n* = 37) of responses as always being an influence on food choices, closely followed by ‘gut comfort’ (28.2%, *n* = 37) and ‘hunger’ (23.7%, *n* = 31) (Figure 1). In regard to doping concern, a greater proportion of never/rarely responses was reported from national and lower competition-level athletes (61.4%, *n* = 43) compared to Olympic (25.7%, *n* = 18) and other international-level athletes (12.9%, *n* = 9; X^2^(2) = 13.1, ϕc = 0.34, *p* = 0.001).

Thirty-four comments were received to the open-ended questions, and of these, only 10 were relevant to the question and not already represented in the AFCQ or the additional 13 items. The responses were coded as: cravings and preferences (*n* = 3), habits (*n* = 2), seasonal foods (*n* = 2), weather (*n* = 2), and sleeping hours (*n* = 1). 

**Figure 2:**
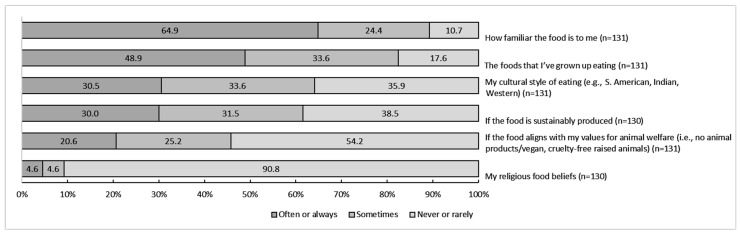
The proportion of 131 athletes rating how frequently (never/rarely, sometimes, and often/always) 6 indicator items from the factors’ ‘usual eating practices’ and ‘food values and beliefs’ influence their food choices.

Figure 2 displays the proportion of responses to items within the ‘usual eating practices’ and ‘food values and beliefs’ factors. Analysis of the items against demographic characteristics identified significant differences for two items. Firstly, animal welfare was more frequently an influence for participants from individual sports (sometimes 30.9%, *n* = 29; never or rarely 46.8%, *n* = 44) compared to participants in team-based sports (sometimes 10.8%, *n* = 4; never or rarely 73.0%, *n* = 27, X^2^(2) = 8.07, ϕc = 0.18, *p* = 0.018, *n* = 131). 

### 3.2. Reliability Results

Reliability scores ranged from 0.60–0.92, with seven factors achieving scores of 0.7 or higher (Table 4). Compared to one or both major competition samples, the online sample received significantly different reliability scores for factors ‘emotional influence’, ‘nutritional attributes of the food’, and the ‘influence of others’. No significant differences between the two previous major competition samples were identified. 

‘Usual eating practices’ and ‘food values and beliefs’ received the lowest reliability scores; however, they did not significantly differ in comparison to the previous values obtained for the major competition samples. Both factors contained a single item that, if removed, would improve their internal consistency. Excluding the item related to religious food beliefs improved α = 0.74 and changed the median to 2.50 for ‘food values and beliefs’. A similar improvement was observed when cultural eating style was excluded from ‘usual eating practices’, α = 0.74 and Mdn = 3.50. The religious item returned both item-to-total and item-to-item correlation scores below the acceptable threshold (Table 4). 

## 4. Discussion

This study examined the application and reliability of the AFCQ when applied as an online questionnaire to athletes outside of a major competition setting. The AFCQ demonstrated acceptable reliability for seven of the nine factors, with ‘usual eating practices’ and ‘food values and beliefs’ receiving the lowest scores. Three factors (emotional influence, nutritional attributes of the food, and influence of others) had significantly lower reliability in this sample compared to one or both previous samples. The results imply that for these three factors, their internal consistency may be affected by the application environment or varying circumstances at the time of data collection. ‘Emotional influence’ was more frequently rated as an influence by injured and ill participants, which may have inflated the consistency the indicator items were rated, in turn increasing the reliability score compared to the previous samples. Additionally, the sample recruitment occurred during 2020 when the global impact of the COVID-19 pandemic affected training, competitions, and most day-to-day activities, which may have heightened the athlete’s emotional state [23,24].

The factors ‘nutritional attributes of the food’ and ‘influence of others’ had lower scores for reliability compared to one or both of the previous major competition samples. Items within these factors may have been slightly more consistent in their effect on food choices when athletes completed the questionnaire in a competition setting. Increased attention to the nutritional attributes of the food may occur with higher-calibre athletes or during competition, when the dietary intake of an athlete may impact on sports performance is more overt [2]. Research with college runners has shown there is increased attention to food choices at critical times in their sport season and then more apathy or indifference regarding the nutritional value of their food choices during the off-season [2]. Differences in responses to the nutritional attributes factor in this study were not detected according to the phase of competition or athlete level. However, athletes who reported receiving most of their nutrition education from a nutrition professional were less frequently influenced by others and more frequently influenced by the nutritional attributes of a food than those obtaining their information from other sources. Advice from an expert such as a sports dietitian can assist athletes in gaining an understanding of individual dietary requirements, food sources of nutrients, healthy eating, and appropriate use of dietary supplements. It is feasible that the nutrition education an athlete has received impacts the importance of the nutritional attributes of the food when choosing what to eat. This may explain the variability of the internal consistency of this factor. Further studies could investigate the impact of a nutrition education intervention on the factors influencing athlete food choice with a particular focus on nutritional attributes of the food.

The reliability score for the ‘influence of others’ was significantly less compared to both previous major competition samples. It is feasible that other athletes (indicator item) may correlate with the items on family and friends more consistently in a competition setting where the presence of athletes is more prominent. Minor differences of reliability in future studies are possible depending on the athlete’s sport type and who they live with. Regardless, the factor still achieved an acceptable reliability score and has sufficient content coverage to yield practical information from athletes in various situations. 

The reliability scores for ‘usual eating practices’ and ‘food values and beliefs’ were not statistically different in comparison to the major competition samples (Table 4), implying that the lower scores are likely not related to the setting. When modelled with one item excluded, namely the item of religious food beliefs (food values and beliefs) and cultural eating style (usual eating practices), both factors improved reliability above the acceptable threshold. These indicator items may receive differing responses depending on the participants’ characteristics (e.g., their personal values), resulting in each sample in a lowered reliability score. Analysis identified one item (animal welfare) in the ‘food value and beliefs’ factor that differed according to participant characteristics. The small effect of this differences is likely not enough to explain the low reliability score. Other characteristics could have impacted the items but were unable to be detected given diversity and size of the sample. Furthermore, the distribution of responses and item correlations for the religious food beliefs item does not support strong internal consistency and provides added justification for excluding the item from this factor. 

There is rationale for the relationship between animal welfare and religious food beliefs, as some religions include dietary restrictions related to animals (e.g., Buddhist, Hindu, Jewish, Muslim, Pagan, Sikh, and Sufi) [25]. Research at the 2010 Commonwealth Games identified that dietary styles were more often based on religious influence for athletes from non-Western regions (e.g., Africa, Sri Lanka, South East Asia, India, and Pacific Islands) compared to those from Western regions [16]. Religious influence may contribute more to the broader food culture in countries categorised as non-Western. In the Universiade sample, a larger proportion of non-Western-region athletes found ‘food values and beliefs’ a more influential factor than their Western-region counterparts [9]. The smaller proportion of participants from non-Western regions in the present sample may explain the lower correlations between the indicator items. 

Unless the AFCQ is applied in populations with a strong religious presence, the religious food beliefs item may warrant separate analysis as an individual item not aligned to a factor. Examining the internal consistency of the ‘food values and beliefs’ factor can help to verify if it is more appropriate to analyse the item separately. Three indicator items are generally warranted for each factor, and thus, further research could investigate whether another item could replace religious food beliefs in ‘food values and beliefs’. Future research exploring this factor would benefit from collecting information on the participants’ religion and strength of belief/practice.

Indicator items within the ‘usual eating practices’ factor did not differ by other groups based on participant characteristics. In the major competition samples, the item-to-item correlations were all acceptable. The present sample is the first instance the item on cultural eating practices has received low item-to-item correlations and the second for a low item-to-total correlation. It is plausible that some factors may have fluctuating reliability due to the AFCQ being comprehensive enough to cover the multifaceted food choice domain while remaining suitable for application with a range of athletes. Repeat applications of the AFCQ with reliability scores reported will provide more evidence to confirm if this is a lower but consistent factor or should be modified or excluded as an item. 

Overall, the relative ratings among the highest (performance) and lowest (food beliefs) influential factors and the most frequently rated additional single-items factors (eating occasions and hunger) were consistent with the descriptive analysis of the major competition samples [9]. Further similarity was found among differences in specific factors relevant to sex and region [9]. ‘Weight control’ was found to differ if the participant had indicated that they were trying to modify their physique. This finding is reassuring in that the ‘weight control’ factor can discern between groups that would be expected to be influenced differently by this factor [9,26]. Interestingly, no differences were found between the phase of competition and the importance of the various factors in the AFCQ. We would expect that ‘performance’ may be of less influence for those out of a competition season, but the relatively small sample size and diversity of the sample may have impacted any potential relationships between these variables. Repeated AFCQs of more homogenous athlete samples would be more effective for investigating any variation across phases of competition. 

A limitation of this study is the potential self-selection bias that the convenience sampling and incentive prize may have introduced. Self-selection bias may have contributed towards the greater representation from female participants and those with a higher education level [27,28]. Disseminating the study via professional networks likely increased representation of participants who valued or studied nutrition and who had previously received nutrition education from professionals. Furthermore, athletes outside the reach of the professional networks would have had less opportunity for participation, impacting recruitment of athletes from all sports, levels, and regions. We also recognise that we grouped athletes based on perceived similarities, e.g., Western/non-Western, but that this might not capture some differences, e.g., cultural diversity. These limitations may reduce the strength with which the findings can be generalized, but they do provide a starting point for work in this area. Future research utilising the AFCQ with a more uniform athlete population would prove useful for examining the reliability and descriptive outputs that may assist in any tailoring necessary for specific sports. Lastly, as the data are self-reported, social desirability bias could have impacted some responses, especially if a coach or nutrition professional had promoted the athlete to participate. However, considering the questionnaire was completed online in the athlete’s own time, and the pre-questionnaire information reassured anonymity, potential bias was likely of minor consequence to the outcomes.

## 5. Conclusions

Overall, the findings support the AFCQ’s reliability when applied under circumstances that better reflect an athlete’s usual setting or food environment. Seven of the nine factors demonstrated acceptable or greater reliability, and two achieved tolerable levels that were not statistically different from the previous samples. The ‘influence of others’ factor had significantly lower reliability in this study, indicating that the communal nature of major competition settings may have a modulating effect on the factor’s internal consistency. Variability in this factor’s reliability may be due to characteristics of the athlete/s, and future applications of the AFCQ should interpret results with consideration to the athletes’ sport, living, and eating situations.

Examination of the factors with lower reliability identified one item from each that, if excluded, would improve reliability. The outcomes of this study suggest that the item ‘religious food beliefs’ may warrant exclusion from the ‘food values and beliefs’ factor due to recurring internal consistency issues. However, in populations where religious practice is prominent, the item may retain its relevancy. Mixed internal consistency results for the cultural eating style item (usual eating practices factor) necessitates more evidence before coming to a conclusive decision. Future applications of the questionnaire are recommended with more homogenous athlete groups with reliability scores published to assist in future reviews and modifications to the AFCQ.

## Figures and Tables

**Table 1:** Summary of data collection variables.

Demographic Characteristics		
Sex Date of birth Country the athlete represents Highest level of education Prior nutrition education ◦ Source of education Language/s spoken at home ◦ English reading and writing confidence (1–10 scale)	Sport and event Highest level of competition Broad phase of competition ◦ Pre-season ◦ In season ◦ Off season ◦ Other (open response)	Goals or circumstances impacting training: ◦ Injury ◦ Illness ◦ Recovery from surgery ◦ Trying to change body weight or shape ◦ Other (open response)
**AFCQ factors**	**Additional single-item factors**	**Specific indicator items**
Emotional influences Food and health awareness Food values and beliefs Influence of others Nutritional attributes of the food Performance Sensory appeal Usual eating practices Weight control	Money Hunger Convenience Eating occasion Eating location Medical conditions Allergies Gut comfort Risk of doping Food availability Busy schedule Sodium content Fibre content	Usual eating practices: ◦ Cultural eating practices ◦ Familiarity ◦ Food grown-up eating Food values and beliefs: Sustainable value ◦ Animal welfare ◦ Religious food beliefs

AFCQ, Athlete Food Choice Questionnaire.

**Table 2:** Participant characteristics of 131 athletes.

Characteristic	*n* (%)	Characteristic	*n* (%)
**Sex**		**Goa** **ls or circumstances impacting current training**	
Male	55 (42.6)	Injured, ill, or recovering from surgery	34 (26.0)
Female	74 (57.4)	Trying to change their body weight/shape	39 (29.8)
**Country region ^a^**		**Sport**	
Africa	11 (8.4)	Ball and net team	32 (24.4)
Asia and India	7 (5.3)	Endurance	54 (41.2)
Latin Americas and Caribbean	9 (6.9)	Power, sprint, and racquet	21 (16.0)
Continental Europe	6 (4.6)	Skill-based	12 (9.2)
North America	37 (28.2)	Aesthetic and weight category	12 (9.2)
Oceania	55 (42.0)		
British Isles	6 (4.6)		
**Highest level of education**		**Broad phase of competitive season ^b^**	
Senior school or less	32 (24.6)	Pre-season	33 (27.0)
Attended or attending tertiary institute	39 (30.0)	In-season	62 (50.8)
Completed undergraduate (bachelor) degree	59 (45.4)	Out of season (off-season)	27 (22.1)
**Source of prior nutrition education**		**Highest level of competition**	
Nutritionist, dietitian, or studied nutrition	82 (63.1)	World competitions	45 (34.4)
Nutritionist or dietitian	66 (50.8)	Olympics (including Youth and Paralympic Games)	8 (6.1)
Studied nutrition at University/College	16 (12.3)	World Championships or World Cup	37 (28.2)
Other sources	48 (36.9)	Other international competitions	24 (18.3)
Coach	14 (10.8)	Commonwealth, Pan America, Asian Games, etc.	14 (10.7)
Doctor	2 (1.5)	Universiade, Collage Games, International meetings	10 (7.6)
Another person	6 (4.6)	National and state competition	62 (47.3)
No nutrition education	24 (18.5)	National competitions	44 (33.6)
Unsure	2 (1.5)	State and local competitions	18 (13.7)

Demographic characteristic headings identified in bold; characteristics collapsed into categories are distinguished with an underline. Missing values: Sex not specified (*n* = 2); nutrition education (*n* = 1); highest level of education (*n* = 1); broad phase of competitive season (*n* = 9). ^a^ Testing region: Western (*n* = 97): North America, British Isles, and Oceania (Australia and New Zealand); and non-Western (*n* = 34): Africa, Asia and India, Latin Americas and Caribbean, Continental Europe, and Oceania (Fiji). ^b^ Participants impacted by injury, illness, or recovery from surgery (*n* = 34) were excluded.

**Table 3:** Median score for the AFCQ factors based on athlete sex, country region, sport, broad competition phase, and nutrition education (*n* = 131).

		*n*	Performance	Food and Health Awareness	Sensory Appeal	Nutritional Attributes of the Food	Weight Control	Usual Eating Practices	Influence of Others	Emotional Influence	Food Values and Beliefs
Total		131	4.00	3.75	3.67	3.60	3.50	3.33	3.00	2.75	2.00
Sex	Female	74	4.00	4.00	4.00	3.80	3.50	3.33	3.00	3.00	2.33
	Male	55	4.33	3.50	3.67	3.60	3.25	3.33	2.83	2.50	2.00
	*p*-value		NS	0.008 *	NS	NS	NS	NS	NS	NS	NS
Country region ^a^	Western	97	4.33	4.00	4.00	3.60	3.50	3.33	3.00	2.75	2.00
	non-Western	34	4.00	3.50	3.67	3.80	3.50	3.33	2.67	3.00	2.00
	*p*-value		NS	0.033 *	0.022 *	NS	NS	NS	NS	NS	NS
Source of nutrition education	Nutritionist ^b^	82	4.33	3.75	3.67	3.80	3.25	3.33	2.67	2.75	2.33
	Other sources	48	4.00	3.50	3.67	3.40	3.50	3.33	3.00	2.75	2.00
	*p*-value		NS	NS	NS	0.005 *	0.026 *	NS	0.033 *	NS	NS
Broad competition phase ^c^	Pre-season	25	4.31	3.66	3.79	3.75	3.27	3.35	2.72	2.65	2.35
	In-season	47	4.17	3.69	3.73	3.55	3.33	3.30	2.83	2.62	2.18
	Off-season	21	4.41	3.52	3.73	3.55	3.24	3.29	2.75	2.35	2.21
	*p*-value		NS	NS	NS	NS	NS	NS	NS	NS	NS
Sport ^d^	Individual	94	4.00	4.00	3.67	3.80	3.50	3.33	3.00	3.00	2.33
	Team	37	4.33	3.50	3.67	3.20	3.00	3.33	2.83	2.25	2.00
	*p*-value		NS	0.015 *	NS	0.013 *	NS	NS	NS	0.007 *	0.032 *
	Ball and net team	32	4.33	3.50	3.67	3.40	3.25	3.33	2.67	2.13	1.83
	Endurance	54	4.17	4.00	3.83	3.80	3.25 ^	3.33	3.00	3.00	2.33
	Power, sprint, racquet	21	4.33	3.75	3.67	3.40	3.25 ^	3.33	3.00	3.00	2.33
	Skill-based	12	4.00	3.50	4.00	3.20	3.50	3.33	3.00	3.25	2.00
	Aesthetic and weight category	12	4.33	4.13	4.00	3.90	4.00 ^	3.50	3.00	2.38	2.67
	*p*-value		NS	NS	NS	NS	0.024 **	NS	NS	NS	NS

NS, not significant (*p* > 0.05). * Mann–Whitney U test statistics: food and health awareness (Sex 2586.5, country region 1245.0, sport 1268.0), sensory appeal (Country region 1220.5), nutritional attributes of the food (nutrition education 1392.5, sport 1254.0), weight control (nutrition education 2428.5), influence of others (nutrition education 2374.0), emotional influences (sport 1217.0), food values and beliefs (sport 1323.50). ** Kruskal–Wallis H test (H(4) = 10.93). ^ Pairwise comparison for weight category against: endurance (*p* = 0.024) and power, sprint (*p* = 0.040). ^a^ Western: North America, British Isles, and Oceania (Australia and New Zealand); non-Western: Africa, Asia and India, Latin Americas and Caribbean, Continental Europe, and Oceania (Fiji). ^b^ Nutrition education from nutritionist, dietitian or studied nutrition at tertiary level. ^c^ Participates recovering from injury, illness, or surgery (*n* = 34), and missing values (*n* = 4) were excluded. ^d^ Individual: equestrian, bowling, diving, figure skating, golf, shooting, weightlifting, wrestling, gymnastics, swimming, cycling, triathlon, surf lifesaving, stand-up paddle board racing, kayaking, canoe, and track and field; team: AFL, cheerleading, hockey, netball, rugby, soccer, volleyball, sailing, lawn bowls, and dragon boating.

**Table 4:** Comparison across three independent samples of the internal consistency scores for nine Athlete Food Choice Questionnaire factors (Cronbach’s alpha coefficients) and item correlation coefficients for two factors.

Factor (Number of Indicator Items) *		Online		Commonwealth Games		Universiade		*p*-Value
		α (CI)	*n*	α (CI)	*n*	α (CI)	*n*	
Emotional influence (4) *		0.92 (0.90–0.94) ^a,b^	130	0.87 (0.84–0.90) ^a^	186	0.83 (0.78–0.87) ^b^	156	0.001
Weight control (4)		0.84 (0.79–0.88)	129	0.76 (0.70–0.81)	186	0.83 (0.78–0.87)	149	NS
Performance (3)		0.81 (0.75–0.86)	130	0.85 (0.81–0.88)	186	0.84 (0.79–0.88)	152	NS
Sensory appeal (3) *		0.80 (0.73–0.85)	131	0.74 (0.67–0.80)	186	0.80 (0.74–0.85)	151	NS
Nutritional attributes of the food (5) *		0.80 (0.74–0.85) ^c^	129	0.88 (0.85–0.91) ^c^	186	0.85 (0.81–0.88)	150	0.033
Food and health awareness (4)		0.74 (0.66–0.81)	131	0.81 (0.76–0.85)	186	0.78 (0.72–0.83)	151	NS
Influence of others (3)		0.70 (0.60–0.78) ^d,e^	130	0.85 (0.81–0.88) ^d^	186	0.85 (0.80–0.89) ^e^	153	0.004
Usual eating practices (3)		0.64 (0.52–0.74)	131	0.66 (0.57–0.74)	186	0.77 (0.70–0.83)	153	NS
Food values and beliefs (3)		0.60 (0.46–0.71)	129	0.62 (0.51–0.71)	186	0.69 (0.59–0.77)	147	NS
**Item-to-total correlations**		**r**	***n***	**r**	***n***	**r**	***n***	
Usual eating practices	Cultural eating practices	0.32	131	0.45	186	0.53	153	N/A
	Familiarity	0.53	131	0.50	186	0.69	153	N/A
	Food grown up eating	0.52	131	0.47	186	0.60	153	N/A
Food values and beliefs	Sustainable value	0.49	129	0.49	186	0.49	147	N/A
	Animal welfare	0.60	129	0.45	186	0.52	147	N/A
	Religious food beliefs	0.20	129	0.35	186	0.53	147	N/A
**Item-to-item correlations ^$^**								
**Usual eating practices**	**Cultural eating practices**	**Familiarity**		**Food values and beliefs**	**Sustainable value**		**Animal welfare**	
Food grown up eating	0.28; 0.36; 0.42	0.58; 0.42; 0.63		Religious food beliefs	0.10; 0.32; 0.43		0.25; 0.28; 0.46	
Familiarity	0.30; 0.40; 0.53	N/A		Animal welfare	0.59; 0.46; 0.40		N/A	

CI, 95% confidence interval; NS, not significant (*p* > 0.05); N/A, not applicable. ^a^ X^2^(1) = 5.15, *p* = 0.023; ^b^ X^2^(1) = 11.78, *p* < 0.001; ^c^ X^2^(1) = 6.90, *p* = 0.010; ^d^ X^2^(1) = 9.25, *p* = 0.002; ^e^ X^2^(1) = 8.30, *p* = 0.004; * The Universiade sample used the 36-item questionnaire, and the number of indicator items were seven for ‘nutritional attributes of the food’, five for ‘emotional influence’, and four for ‘sensory appeal’. ^$^ Correlations presented in the order Online, Commonwealth Games, Universiade.

## Data Availability

The data presented in this study are available on request from the corresponding author. The data are not publicly available due to privacy reasons.

## Supplementary Materials

The following supporting information can be downloaded at: https://www.mdpi.com/article/10.3390/ijerph19169981/s1.
