# Supplementary material for: Reliability of the Athlete Food Choice Questionnaire in Diverse Settings
Source: Int J Environ Res Public Health. 2022 Aug 12;19(16):9981. doi: 10.3390/ijerph19169981 (PMC9407750; doi:10.3390/ijerph19169981)
Supplement: Supplementary file 1 [file ijerph-19-09981-s001.zip › ijerph-1791774-supplementary.pdf]

Supplementary

Formula and calculations for estimating the minimum sample size needed to compare Cronbach Alpha scores.

$$\delta = \frac{1 - CA_1}{1 - CA_2}$$

$$n = \left[\frac{2\left(\frac{k_1}{k_1 - 1} + \frac{k_2}{k_2 - 1}\right)(Z_{\alpha/2} + Z_\beta)^2}{In(\delta)^2} \right] + 2$$

$$\delta = \frac{1 - 0.80}{1 - 0.90}$$

$$\delta = 2.00$$

$$\delta = \frac{1 - 0.65}{1 - 0.80}$$

$$\delta = 1.75$$

$$n = \left[\frac{2\left(\frac{3}{3 - 1} + \frac{3}{3 - 1}\right)(1.960 + 0.841)^2}{In(2.00)^2} \right] + 2$$

$$n = 100$$

$$n = \left[\frac{2\left(\frac{3}{3 - 1} + \frac{3}{3 - 1}\right)(1.960 + 0.841)^2}{In(1.75)^2} \right] + 2$$

$$n = 152$$

$$n = \frac{100+152}{2} = 126$$
